# Unraveling the Anti-Aging Properties of Phycocyanin from the Cyanobacterium Spirulina (*Arthrospira platensis*)

**DOI:** 10.3390/ijms25084215

**Published:** 2024-04-11

**Authors:** Mariachiara Nova, Stefania Citterio, Enzo Martegani, Sonia Colombo

**Affiliations:** Department of Biotechnology and Biosciences, University of Milano-Bicocca, Piazza della Scienza 2, 20126 Milan, Italy; m.nova@campus.unimib.it (M.N.); stefania.citterio@unimib.it (S.C.); enzo.martegani@unimib.it (E.M.)

**Keywords:** ROS, aging, chronological life span, yeast, *S. cerevisiae*

## Abstract

In recent years, marine natural products have become one of the most important resources of novel lead compounds for critical diseases associated with age. *Spirulina*, a dietary supplement made from blue-green algae (cyanobacteria: scientific name *Arthrospira platensis*), is particularly rich in phycocyanin, a phycobiliprotein, which accounts for up to 20% of this cyanobacterium’s dry weight and is considered responsible for its anti-cancer, anti-inflammatory and antioxidant activities. Although the anti-aging activity of phycocyanin has been investigated, how exactly this compound works against aging remains elusive. The aim of our research is to use the yeast *Saccharomyces cerevisiae* as a model organism to investigate the anti-aging properties of phycocyanin from *A. platensis*. Our results show that phycocyanin has a powerful anti-aging effect, greatly extending the chronological life span of yeast cells in a dose-dependent way, as the effect was also pronounced when cells were grown in SD medium under calorie restriction conditions (0.2% glucose). Both ROS and accumulation of dead cells were followed by staining chronologically aged cells with dihydrorhodamine 123 (DHR123) and propidium iodide (PI). Interestingly, we found that most of the aged phycocyanin-treated cells, which were unable to form colonies, were actually ROS+/PI–. Finally, we show that the moment in which phycocyanin is added to the culture does not substantially influence its effectiveness in counteracting chronological aging.

## 1. Introduction

The major health problems observed all over the world are often associated with age since people are living longer [1]. In recent years, marine natural products have become one of the most important sources of new compounds potentially useful in the treatment of age-related diseases, such as cancer, neurodegenerative and cardiovascular diseases, metabolic syndrome and many others [2,3,4,5]. *Spirulina* (cyanobacteria: *Arthrospira platensis*) is a photosynthetic Gram-negative filamentous bacterium that grows naturally in alkaline, brackish, marine and fresh waters [6], but it is also commercially produced in large outdoor or greenhouse ponds under controlled conditions [7]. In recent years, *A. platensis* has attracted more and more attention as a potential source of pharmaceutical compounds. Indeed, this cyanobacterium is a source of many but only partially identified and purified compounds with potential health benefits and activities. It is particularly rich in proteins (65% to 70%), as well as phycocyanin (PC), vitamins, beta-carotene, polyunsaturated fatty acids and minerals, especially iron, polysaccharides and others [8]. Phycocyanin, which accounts for up to 20% of this cyanobacterium’s dry weight, is the association of proteins of the phycobiliprotein family and water-soluble pigments of photosynthesis, the phycocyanobilins. Phycocyanin consists of α and β subunits with a molecular weight of 16.3 kD and 18.9 kD, respectively [9]. The determination of the purity of phycocyanin is based on the absorbance ratio A620/A280; the absorbance at 620 nm corresponds to the phycocyanin present in the preparation (it is proportional to the quantity of pigment), while the absorbance at 280 nm corresponds to the total proteins (it is proportional to the proteins in the solution). When A620/A280 is >0.7, phycocyanin is considered edible; when 0.7 ≤ A620/A280 ≤ 3.9, the phycocyanin is considered to be reagent grade; and, finally, when A620/A280 is ≥4.0, the phycocyanin is considered to be analysis level [10]. Phycocyanin is considered responsible for the *A. platensis* anti-cancer, anti-inflammatory and antioxidant activities [11,12,13]. In particular, phycocyanin has been shown to reduce oxidative stress in both yeast and mammals [14,15]. Moreover, data in the literature showed that the antioxidant properties of phycocyanin might arise from both radical scavenging and metal chelation [16]. Since ROS (Reactive Oxygen Species) are considered important factors causing aging, the anti-aging activity of phycocyanin has been investigated. However, the question of how exactly phycocyanin works against aging remains elusive. In particular, the intracellular mechanism of the action of PC has been poorly studied and most of its possible targets are still unclear [9,17,18,19,20].

Senescence (i.e., aging; not to be confused with replicative or cellular senescence) is the main cause of disease and death in the modern world. Consequently, understanding the biological mechanisms of senescence and how they give rise to diseases associated with age, including cardiovascular disease, many forms of cancer, dementia, and many others, is crucial. According to one of the most cited papers in the field of aging published in 2013 [21], the process of aging results from nine causes: genomic instability, telomere attrition, epigenetic alterations, loss of proteostasis, deregulated nutrient sensing, mitochondrial dysfunction, cellular senescence, stem cell exhaustion and altered intercellular communication. However, since then, several theories have been postulated with the aim of explaining aging, which, in synthesis, is a multifactorial process that includes both genetic and environmental factors and intricate pathways and networks (see the following reviews for a detailed description [22,23]). In this context, the yeast *Saccharomyces cerevisiae* is a model organism for eukaryotic cells and can provide significant insights into the human biology of aging, since the principal pathways are conserved between yeasts and higher eukaryotes during evolution [21,24,25,26]. The two approaches to studying aging in *S. cerevisiae* are the chronological life span (CLS) and the replicative life span (RLS). The chronological life span is the approach we used; it is a measure of the mean and maximum survival time of non-dividing yeast populations and it can serve as a system to model aging in post-mitotic mammalian cells [24].

In this paper, we used the yeast *S. cerevisiae* as a model organism to study the anti-aging properties of PC purified from the cyanobacterium *A. platensis*. We show that phycocyanin has a powerful anti-aging effect, greatly extending the chronological life span of yeast cells grown in SD medium, under both calorie restriction (CR) conditions (0.2% glucose) or non-CR conditions (2% glucose). Interestingly, we show an increase in ROS level, which does not correlate with cell death measured by counting the colony-forming units over time, when cells are grown in the presence of phycocyanin. This result might suggest that phycocyanin promotes longevity by inducing hormesis [27,28,29,30], an adaptive response to a variety of stresses according to which low doses of ROS act as essential signalling molecules to promote metabolic health and longevity.

## 2. Results and Discussion

### 2.1. Phycocyanin Delays the Chronological Aging of Yeast Cells

In this study, we used the yeast *Saccharomyces cerevisiae* to investigate the ability of phycocyanin to extend the chronological life span (CLS). In particular, we used phycocyanin kindly provided by Algavista (for more details, see the Materials and Methods section), which was also used by Macedo et al. to demonstrate that this compound protects against α-synuclein toxicity in yeast [31]. We first compared the molecular weight and purity of the phycocyanin used in this study with that of a standard available reagent (Sigma-Aldrich, St. Louis, MO, USA). For this purpose, we performed a polyacrylamide gel electrophoresis in the presence of sodium dodecyl sulphate (SDS-PAGE) and absorbance spectra. Identical patterns were observed for both samples, confirming the quality of the phycocyanin used (Supplementary Figure S1A,B). The quality of used phycocyanin is also demonstrated by the good A620/A280 ratio (3.99) observed. We also determined the stability of the phycocyanin used in this study by analysing the absorbance spectrum both in water and in a SD medium containing 2% glucose, both after preparation (0 h) and after about 10 days at 30 °C. The similar spectra confirmed the stability of the phycocyanin, especially in the SD medium, which is the medium we used during chronological aging experiments (Supplementary Figure S1C).

**Figure 1:**
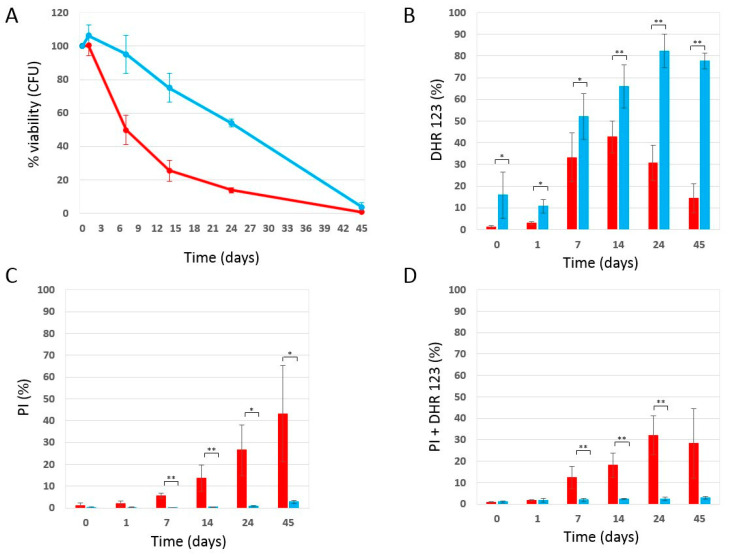
Cell survival and oxidative stress in wild-type W303-1A cells grown in SD medium containing 0.2% glucose in the presence and absence of phycocyanin (given at the moment of inoculation). (**A**) Cell viability of W303-1A cells either untreated (red) or treated (blue) with 4 mg/mL (±0.2 mg/mL) phycocyanin was analysed by measuring colony-forming units (CFU) after 3 days of growth at 30 °C. Cell survival is expressed as % to the CFU at time zero. (**B**) ROS accumulation in W303-1A cells either untreated (red bars) or treated (blue bars) with 4 mg/mL (±0.2 mg/mL) phycocyanin. (**B**–**D**) Flow cytometry analysis of double-stained (DHR 123 and PI) W303-1A cells, either untreated (red bars) or treated (blue bars) with 4 mg/mL (±0.2 mg/mL) phycocyanin. Dihydrorhodamine 123 (DHR 123) was used to assay ROS accumulation, while propidium iodide (PI) was used to stain dead cells (for more details, see Supplementary Figures S2 and S3). The means of 3 independent experiments with standard deviations are reported. Student’s *t*-test * *p* < 0.05 and ** *p* < 0.01.

**Figure 2:**
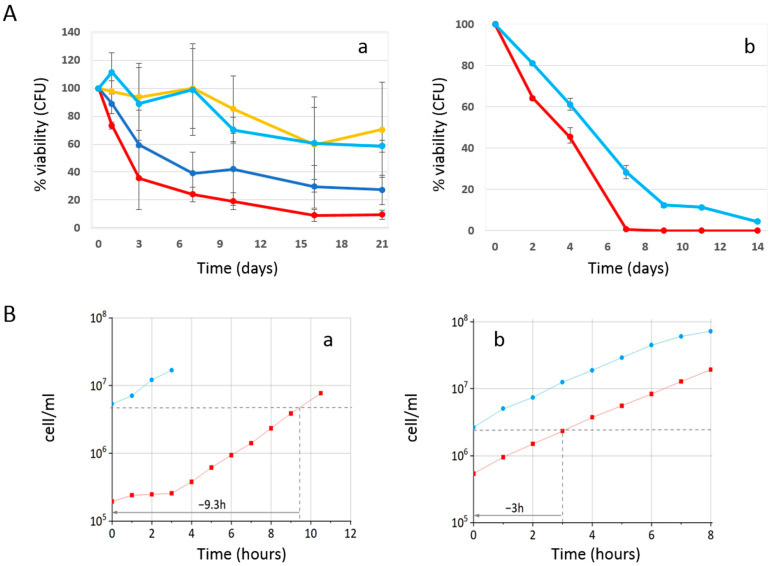
Phycocyanin antagonizes cell chronological aging in a dose-dependent way and is effective even in the presence of 2% glucose. (**A**) Cell survival of wild-type W303-1A cells was monitored with colony-forming units (CFU) during 21 days CLS in SD medium containing 0.2% glucose in the presence of the following concentrations of phycocyanin (given at the moment of inoculation): 0 mg/mL (red line), 1 mg/mL (blue line), 2.3 mg/mL (yellow line), 4.8 mg/mL (light blue line). The means of 3 independent experiments with standard deviations are reported (**a**). Cell survival of wild-type W303-1A cells was monitored with colony-forming units during 14 days CLS in SD medium containing 2% glucose in the presence of 4 mg/mL phycocyanin (given at the moment of inoculation) (**b**). (**B**) Growth kinetic of W303-1A cells in the absence and presence of 4 mg/mL phycocyanin. Cells were inoculated in SD medium containing either 0.2% glucose (**a**) or 2% glucose (**b**) at 30 °C and grown to early exponential phase. Cell density (log scale; cells/mL) was plotted against time. A representative result of two independent experiments is shown.

Initially, we inoculated wild-type W303-1A yeast cells (day 3 of the experiment) in SD medium containing 0.2% glucose (condition of calorie restriction, which is known to increase longevity), in the absence and presence of 4 mg/mL (±0.2 mg/mL) phycocyanin. As mentioned in the Materials and Methods section, given the difficulty in filtering the culture medium containing phycocyanin, 4 mg/mL (±0.2 mg/mL) turned out to be the highest testable concentration. When the glucose present in the culture medium is completely consumed, cells enter the stationary phase, do not carry out any cell division, reach the maximum cell concentration (day 0 CLS) [32], and acquire greater resistance to stress [33]. We monitored the progressive loss of viability of these stationary phase cells kept in their culture medium from day 0 until day 45 and used them as an index of chronological aging [32] (Figure 1). Strikingly, phycocyanin showed a potent anti-aging activity. Indeed, it strongly prolonged the chronological life span of yeast cells with a long-lasting effect over time. In fact, on day 7 of CLS, almost the entire cell population treated with phycocyanin was viable, while the viability of the untreated cell population (control) was approximately 50%. On day 14 of CLS, a viability of 75% and 26% was observed for the population treated with phycocyanin and for the control, respectively. Moreover, on day 25 of CLS, when less than 20% of viable cells were detectable within the control cell population, the population treated with phycocyanin showed a viability of almost 60%. To investigate whether the potent anti-aging activity of phycocyanin was still detectable at lower doses of this compound, W303-1A cells were inoculated (day 3 of the experiment) in SD medium containing 0.2% glucose with variable concentrations of phycocyanin (0–4.8 mg/mL). Indeed, phycocyanin strongly prolonged CLS, acting in a concentration-dependent manner until an apparent saturation effect at dosages higher than 2.3 mg/mL (Figure 2A(a)). Finally, we performed a preliminary experiment to investigate whether the potent anti-aging activity of phycocyanin was still detectable when cells were grown in an SD medium containing 2% glucose, a condition where cells aged very fast [34]. Our results showed that also under this unfavourable condition (i.e., in non-calorie restriction condition), phycocyanin still prolonged the chronological life span of yeast cells (Figure 2A(b)).

In order to investigate the effect of phycocyanin on the growth rate, we evaluated the cell growth in the absence and presence of phycocyanin, using a cell counter (Coulter Counter Z2); in parallel, using the same instrument, we evaluated the average cell volume. In particular, cells of wild-type W303-1A were inoculated in a SD medium containing either 0.2% glucose or 2% glucose in the absence and presence of 4 mg/mL phycocyanin and grown at 30 °C overnight. The following morning, for each treatment, we determined the cell density and the mean cell volume over time. Our results (Figure 2B) showed that both under calorie restriction (CR) conditions (0.2% glucose) or non-CR conditions (2% glucose), the presence of phycocyanin in the medium did not influence the growth rate, which was, on average, a doubling time of 1.5 h. A 10% decrease in the mean cell volume of exponentially growing cells was observed in the presence of phycocyanin, under both tested conditions (0.2% and 2% glucose). In particular, in 0.2% glucose, the mean size of the cells was about 54 and 49 femtoliters in the absence and presence of phycocyanin respectively, while in 2% glucose, the mean size of the cells was about 57 and 52 femtoliters in the absence and presence of phycocyanin, respectively. Interestingly, under both tested conditions (0.2% and 2% glucose), the presence of phycocyanin in the culture medium determined an early entry of cells into the exponential phase of growth, which was more pronounced when the glucose concentration in the culture medium was low.

### 2.2. Effect of Phycocyanin on Reactive Oxygen Species Accumulation

Oxidative stress and the accumulation of reactive oxygen species (ROS) are known to play a key role in aging [35]; moreover, data in the literature attribute an antioxidant role to phycocyanin [11,12]. Therefore, we decided to determine the accumulation of ROS in the context of CLS experiments. As described previously (Figure 1A), we inoculated W303-1A cells in the absence and presence of 4 mg/mL (±0.2 mg/mL) phycocyanin in a SD medium containing 0.2% glucose, determined its ability to form colonies over time and showed that the presence of phycocyanin in the medium significantly decreased the chronological aging of yeast cells. In parallel, using a cytofluorimeter, we determined both the accumulation of ROS and the accumulation of dead cells by staining the chronologically aged cells with dihydrorhodamine123 (DHR 123) and propidium iodide (PI) (Supplementary Figure S2). The results obtained from the cytofluorimetric analysis conducted on the control cells (untreated) are congruent with those obtained in the CLS experiment. The cells, over time, accumulate ROS (Figure 1B,D), lose membrane integrity and die (Figure 1C,D); in particular, a fraction of cells die accumulating ROS (Figure 1D), while a numerically similar fraction die without accumulating reactive oxygen species (Figure 1C; in Supplementary Figure S3, a flow cytometry analysis representative of the DHR123/PI staining is shown). Congruently, the percentage of viable cells, measured by counting the colony-forming units, decreases over time.

On the other end, cells treated with phycocyanin, while being longer-lived than untreated cells (Figure 1A), show a higher level of ROS than the untreated cells themselves (Figure 1B), suggesting that phycocyanin might favour survival by enhancing the level of ROS. Congruently, the percentage of dead (or PI-positive) cells, measured by staining chronologically aged cells with propidium iodide (PI) is very low (Figure 1C,D). This result suggests that a large fraction of the cells treated with phycocyanin still maintain membrane integrity, even when they become unable to form colonies on Petri dishes containing rich medium, and that, actually, an increase in ROS level favours survival. This apparent contrast might be explained by an adaptive response to a variety of oxidative and other stresses called hormesis, which makes cells resistant to higher and normally harmful doses of the same stressing agent. According to this hypothesis, which is supported by a number of experimental data, low doses of ROS act as essential signalling molecules to promote metabolic health and longevity [27,28,29].

### 2.3. Effect of Phycocyanin Added on Day 0 of CLS, When Maximum Cell Density Is Reached

**Figure 3:**
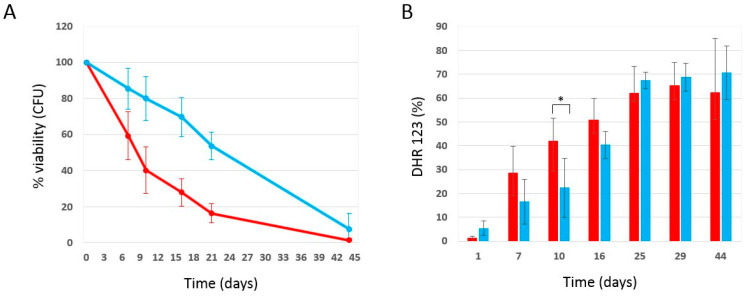
Cell survival and oxidative stress in wild-type W303-1A cells grown in SD medium containing 0.2% glucose in the presence and absence of phycocyanin (given 3 days after inoculation). (**A**) Cell viability of W303-1A cells either untreated (red) or treated (blue) with 0.9 mg/mL (±0.32 mg/mL) phycocyanin was analysed by measuring colony-forming units (CFU) after 3 days of growth at 30 °C. Cell survival is expressed as % to the CFU at time zero. (**B**) ROS accumulation in W303-1A cells either untreated (red bars) or treated (blue bars) with 0.9 mg/mL (±0.32 mg/mL) phycocyanin. Dihydrorhodamine 123 (DHR123) was used to assay ROS accumulation. The means of 3 independent experiments with standard deviations are reported. Student’s *t*-test * *p* < 0.05.

Data in the literature [36] show that the anti-aging effect of drugs can be more or less pronounced depending on the growth phase of the organism at the time the drug is added. In order to investigate this aspect, the chronological aging experiment was repeated by adding phycocyanin to cells of the W303-1A strain on day 0 of the chronological aging experiment, i.e., three days after inoculation, when the maximum cell density was reached, to study whether the moment in which phycocyanin is added influences its effectiveness in counteracting chronological aging. In particular, we inoculated wild-type W303-1A in SD medium containing 0.2% glucose and, on day 3 after inoculation, the culture was divided into two sterile tubes, which were centrifuged in order to separate cell pellets from the culture media (supernatants). Subsequently, phycocyanin was dissolved in one of the two supernatants at a concentration of 5 mg/mL. Both supernatants were then filtered using PES filters (0.22 µm) and the corresponding cell pellet was resuspended in them. Both cultures were incubated at 30 °C and their viability was monitored through the quantification of the CFU and used as an index of chronological aging. As mentioned in the Materials and Methods section, the concentration of phycocyanin after filtration was approximately 20% of the phycocyanin initially dissolved in the exhausted medium. Consequently, the actual concentration was 0.9 mg/mL (±0.32 mg/mL). Strikingly, phycocyanin added at the end of the growth phase (3 days after inoculation) showed, again, a potent anti-aging activity (Figure 3A), comparable with that obtained previously (Figure 1A), by adding 4 mg/mL (±0.2 mg/mL) of phycocyanin at the time of inoculation. In particular, our results (Figure 3A) show that on day 16 of CLS, 70% of the cell population treated with phycocyanin was viable, while the viability of the untreated cell population (control) was less than 30%. On day 21 of CLS, a viability of 54% and 16% was observed for the population treated with phycocyanin and for the control, respectively. In conclusion, considering that the concentration of phycocyanin added 3 days after inoculation was approximately 4 times less than the concentration of phycocyanin added at the time of inoculation, our results indicate that phycocyanin added at the end of the growth phase has a more marked anti-aging effect when compared to that obtained by adding phycocyanin at the time of inoculation. In parallel, using the cytofluorimeter, we determined the accumulation of reactive oxygen species by staining the chronologically aged cells with dihydrorhodamine123 (DHR 123) (Figure 3B). Our results showed that the production of ROS was lower or comparable for phycocyanin-treated cells compared to untreated cells. These data suggest that the addition of phycocyanin at the end of the growth phase might favour survival through a mechanism other than hormesis, previously proposed to explain how an increase in ROS might, in the case of phycocyanin added at the time of inoculation, favour survival. It might be that phycocyanin added on day 0 of CLS (when maximum cell density is reached) acts on signalling mechanisms, as observed for hydroxycitric acid, a caloric restriction mimetic we previously investigated [37]. Genetic analysis on yeast CLS mutants indicated that part of the powerful anti-aging effects of hydroxycitrate can be sensed by Sch9 and Ras2, two conserved key regulators of nutritional and stress signal pathways of primary importance in *S. cerevisiae* [37]. However, it could not be excluded that the reduced ROS level observed when PC was added at the end of the growth phase could be simply explained by the lower concentration of PC present in the medium.

## 3. Materials and Methods

### 3.1. Yeast Strains and Media

The strain used in this study: *Saccharomyce cerevisiae* W303-1A (MATa ade2-1 can1-100 his3-11,15 leu2-3112 trp1-1 ura3-1) [38]. Synthetic-defined media (SD) contained either 0.2% glucose or 2% glucose, 6.7 g/l YNB w/o amino acids (supplied by ForMedium™, Swaffham, UK) and the selective drop-out CSM (Complete Supplement Mixture, supplied by ForMedium™, Swaffham, UK) -HIS -LEU -TRP -URA; in addition, 100 mg/L adenine and 50 mg/L histidine, leucine, tryptophan and uracil were added. Culture density was measured with a Coulter Counter (Coulter mod. Z2) on mildly sonicated, diluted samples. The growth rate was calculated by the increase in cell number/mL at discrete time intervals and by plotting the log of cell concentration as a function of time. The mean cell size was calculated by the cell size distributions acquired during the exponential phase of growth [39]. YEPDA plates contained 2% *w v*^−1^ glucose, 2% *w v*^−1^ peptone, 1% *w v*^−1^ yeast extract, 100 mg/L adenine and 2% *w v*^−1^ agar.

Organic Spirulina Extract (phycocyanin powder), together with a certificate of analysis where it was reported that the percentage of purity was higher than 40%, was kindly provided by Algavista, Chennai, India. However, the filtration step described below allowed us to obtain phycocyanin at a good level of purity, as demonstrated by the result obtained by the SDS-PAGE and by the absorbance ratio A620/A280 (3.99), which was even higher than that obtained for a standard reagent from Sigma (3.69) (Supplementary Figure S1). Phycocyanin powder (phycocyanin in the Results and Discussion section) was dissolved in SD medium (the concentration varies among experiments) and sterilized by filtration through 0.22 μm PES filters. Given the difficulty in filtering the culture medium containing phycocyanin (difficulty, which was more pronounced when phycocyanin was dissolved in exhausted medium compared to fresh medium), and since a deposit of material on the filter was observed during the filtration step, a calibration curve was set up in order to determine whether phycocyanin loss occurred during this step and to determine the quantity of phycocyanin actually present in the medium (Supplementary Figure S4). On average, the concentration of phycocyanin after filtration was approximately 80% of the phycocyanin dissolved in the fresh medium, and about 20% of the phycocyanin dissolved in the exhausted medium. The pH of the fresh medium was 5.7 regardless of the presence of phycocyanin.

### 3.2. Aging Experiments and Cell Viability

We used one of the established procedures to measure chronological life span (CLS), as described by Fabrizio and Longo [32]. The yeast cells were grown in a SD medium (containing either 0.2% glucose or 2% glucose) at 30 °C on a shaker at 160 rpm and prolonged incubation in their original exhaust medium. Chronological aging was monitored after arrest into the stationary phase (day 0 of CLS) as progressive loss of cell viability. In particular, W303-1A cells were plated on YEPDA agar plates in triplicate and colony-forming units (CFUs) were used to monitor viable cells after 3 days of growth at 30 °C.

### 3.3. Dihydrorhodamine 123 (DHR123) and Propidium Iodide (PI) Staining

ROS were detected with DHR123 (Sigma-Aldrich), essentially as described by Madeo et al. [40]. DHR123 was added directly to the culture medium at the final concentration of 5 µg/mL (from a 2.5 mg/mL stock solution in ethanol). After 2 h incubation at 30 °C with shaking, PI (Fluka) was added at the final concentration of 3 µg/mL (from 50 µg/mL in 10 mM of Tris-HCl pH 7.0 stock solution) and incubation was prolonged for an additional 15 min in the dark at room temperature. At the end of this period, cells were diluted in 50 mM Tris-HCl pH 7.5 to 10^7^ cells/mL and analysed using a cytofluorimeter (CytoFLEX©, Beckman Coulter, Inc., Brea, CA, USA). A total of 20,000 events were acquired for each sample and data were processed using CytExpert software (Version 2.6).

### 3.4. Phycocyanin Characterisation

Sodium dodecyl sulphate (SDS)-PAGE (polyacrylamide gel electrophoresis) was employed to analyse the purity of phycocyanin used in this work. A phycocyanin standard (Sigma-Aldrich, St. Louis, MO, USA) was used as a reference. The polypeptide analysis was performed in a 12% SDS-PAGE in pH 8.8 Tris-HCl buffer and a stacking gel of 4% in pH 6.8 Tris-HCl. Tris-Gly buffer (pH 8.6) containing 0.1% (*w*/*v*) SDS was used as electrode buffer. The gel was stained with EZBlue Staining Reagent (Sigma-Aldrich). The absorbance spectrum of phycocyanin was determined by measuring the absorbance between 200 nm and 800 nm (Jasco V-770 spectrophotometer), in order to compare the extract with the standard and to determine its stability. The purity of the phycocyanin and the standard was determined by the ratio between the absorbance at 620 nm (A620), proportional to the amount of pigment, and the absorbance at 280 nm (A280), proportional to the proteins in the solution [10]. The phycocyanin and standard presented a purity ratio of 3.99 and 3.69, respectively (Supplementary Figure S2B).

### 3.5. Statistical Analysis

All the experiments were conducted at least in triplicate unless otherwise indicated, and the mean and standard deviation were shown. The Student’s *t* test was used for assessing the significance of the experimental data. The experimental data were elaborated with Excel TM.

## 4. Conclusions

Since *Saccharomyces cerevisiae* is a useful model organism for studying aging and aging-associated diseases, we used this yeast to study the anti-aging properties of phycocyanin purified from the cyanobacterium *A. platensis*. Phycocyanin has been shown to reduce oxidative stress in both mammals and the aerobic yeast *Yarrowia lipolytica* [14,15]. Interestingly, we show that cells treated with phycocyanin at the time of inoculation, while being longer-lived than untreated cells, show a higher level of ROS than the untreated cells themselves, suggesting that phycocyanin might favour survival by enhancing the level of ROS in *S. cerevisiae*. A possible interpretation of this result is that phycocyanin might promote longevity by inducing hormesis, an adaptive response to a variety of oxidative and other stresses [27,28,29,30]. On the other hand, we also show that phycocyanin added at the end of the growth phase (3 days after inoculation) showed again a potent anti-aging activity, but the production of ROS was lower or comparable for phycocyanin-treated cells compared to untreated cells, suggesting that the addition of phycocyanin at the end of the growth phase might favour survival through a mechanism other than hormesis, or that phycocyanin does not act as an antioxidant under our experimental conditions. We suggest that, in this case, phycocyanin might act on signalling mechanisms, as observed for hydroxycitric acid, a caloric restriction mimetic we previously investigated [37]. Moreover, to support this hypothesis, data in the literature show that in mammalian cells phycocyanin modulates AKT and AMPK signalling, ameliorating the senescence of mesenchymal stem cells and protecting against high-glucose and high-fat diet-induced diabetes in mice, respectively [18,19]. In order to investigate whether phycocyanin might act on signalling mechanisms, in future work, we will test the effect of phycocyanin on mutants bearing deletion in one of the signalling pathways connected to longevity regulation in yeast, such as mutants in the Ras-cAMP or the Tor-Sch9 pathways [34]. However, the reduced level of ROS observed when phycocyanin was added at the end of the growth phase could be simply explained by the lower concentration of this compound present in the medium, due to the difficulty in filtering the culture medium containing phycocyanin when this compound was dissolved in exhaust medium.

## Data Availability

The data that support the findings of this study are available in the figures and Supplementary Material of this article.

## Supplementary Materials

The following are available online at https://www.mdpi.com/article/10.3390/ijms25084215/s1.
