# Supplementary material for: Unraveling the Anti-Aging Properties of Phycocyanin from the Cyanobacterium Spirulina (Arthrospira platensis)
Source: Int J Mol Sci. 2024 Apr 11;25(8):4215. doi: 10.3390/ijms25084215 (PMC11050328; doi:10.3390/ijms25084215)
Supplement: Supplementary file 1 [file ijms-25-04215-s001.zip › ijms-2926340-supplementary.pdf]

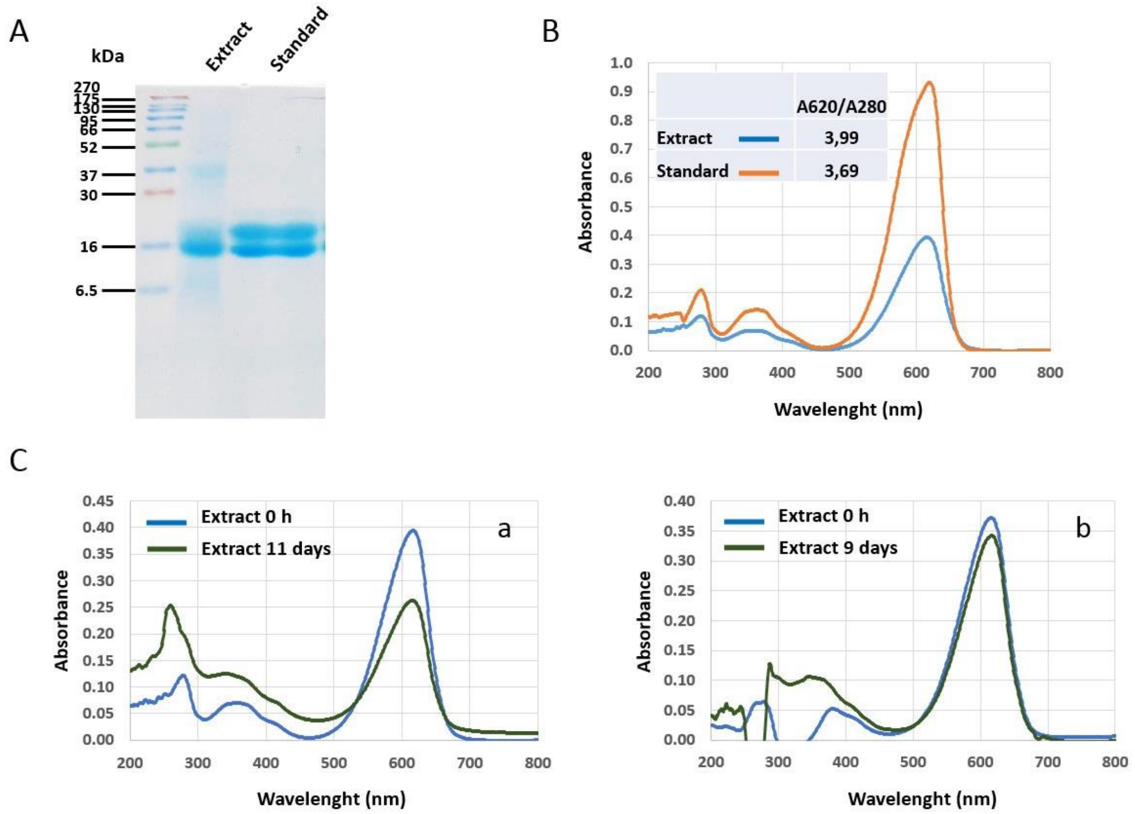


**Supplementary Figure S1:** Phycocyanin characterization. (A) Polyacrylamide gel electrophoresis under denaturing conditions (SDS-PAGE, 12%).  50µg of phycocyanin used in this study (Extract) and >50µg of the Sigma standard (Standard) were loaded into the wells. The gel was stained with EZBlue Staining Reagent (Sigma-Aldrich). (B) Determination of the purity of phycocyanin used in this study. The degree of purity is determined by the A620/A280 ratio of samples prepared at a concentration of 100µg/mL in water. The absorption spectrum of the phycocyanin used in the study (Algavista, in blue) and of the Sigma commercial standard (in orange) is shown. (C) Determination of the stability of phycocyanin used in this study. Stability is determined by reading the A620 absorbance of phycocyanin dissolved in water (a) and in a SD medium containing 2% glucose (b), at the time of preparation and after about 10 days of incubation at 30°C. The figure also shows the absorption spectra of the extract at the time of preparation (blue) and after about 10 days incubation at 30°C (green).

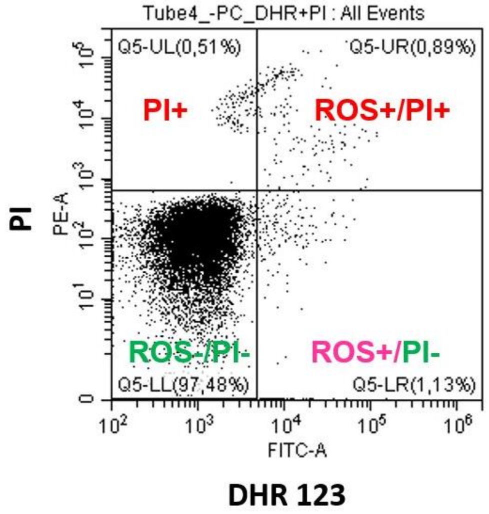


**Supplementary Figure S2:** Flow cytometry analysis of double stained (DHR 123 and PI) yeast cells. DHR 123 fluorescence is shown on the X-axis, while PI fluorescence is shown on the Y-axis. Cells in the lower left quadrant represent cells negative for both stains (DHR 123-/PI-) and therefore the viable population. Cells in the lower right quadrant represent DHR 123+/PI- cells, i.e. live cells with high level of ROS. In the upper left quadrant, PI+ cells are shown that die without accumulating ROS. Cells in the upper right quadrant represent DHR 123+/PI+ cells, i.e. dead cells with accumulation of ROS.

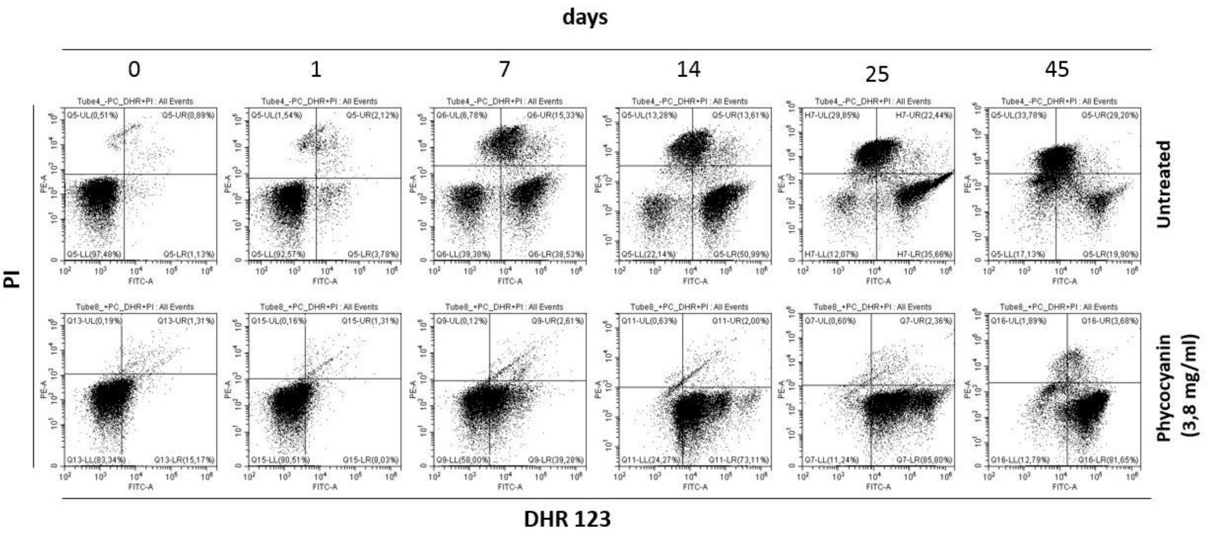


**Supplementary Figure S3:** Flow cytometry analysis of double stained (DHR 123 and PI) yeast cells. DHR 123 fluorescence is shown on the X-axis, while PI fluorescence is shown on the Y-axis. Cells in the lower left quadrant represent cells negative for both stains (DHR 123-/PI-) and therefore the viable population. Cells in the lower right quadrant represent DHR 123+/PI- cells, i.e. live cells with high level of ROS. In the upper left quadrant, PI+ cells are shown that die without accumulating ROS. Cells in the upper right quadrant represent DHR 123+/PI+ cells, i.e. dead cells with accumulation of ROS.

A

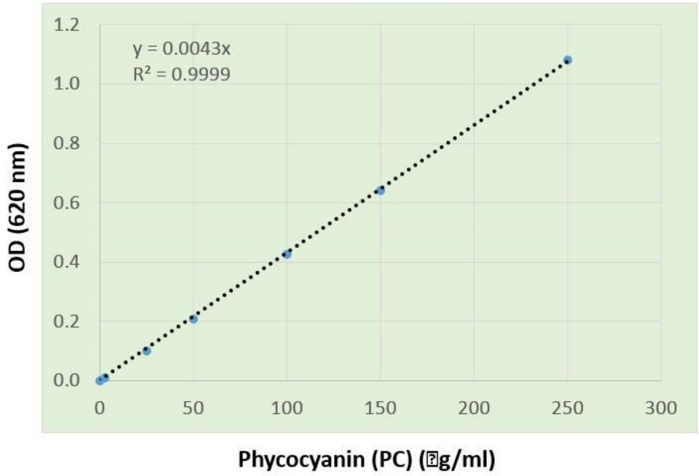


B

| PC (µg/mL) | OD(620nm) | OD(620nm) | Average |
|---|---|---|---|
| 0 | -0,002 | 0,003 | 0,000 |
| 0,25 | -0,001 | 0,000 | -0,001 |
| 2,5 | 0,008 | 0,009 | 0,008 |
| 25 | 0,099 | 0,103 | 0,101 |
| 50 | 0,207 | 0,211 | 0,209 |
| 100 | 0,427 | 0,428 | 0,427 |
| 150 | 0,641 | 0,638 | 0,639 |
| 250 | 1,080 | 1,081 | 1,081 |

**Supplementary Figure S4**: (A) Calibration curve. (B) Starting from a 250 µg/mL phycocyanin stock solution, dilutions were performed (in duplicate) according to the reported scheme and absorbance was read at wavelength ʎ=620nm.
